# A pathway-centric approach to rare variant association analysis

**DOI:** 10.1038/ejhg.2016.113

**Published:** 2016-08-31

**Authors:** Tom G Richardson, Nicholas J Timpson, Colin Campbell, Tom R Gaunt

**Affiliations:** 1 MRC Integrative Epidemiology Unit, School of Social and Community Medicine, University of Bristol, Bristol, UK; 2 Intelligent Systems Laboratory, University of Bristol, Bristol, UK

## Abstract

Current endeavours in rare variant analysis are typically underpowered when investigating association signals from individual genes. We undertook an approach to rare variant analysis which utilises biological pathway information to analyse functionally relevant genes together. Conventional filtering approaches for rare variant analysis are based on variant consequence and are therefore confined to coding regions of the genome. Therefore, we undertook a novel approach to this process by obtaining functional annotations from the Combined Annotation Dependent Depletion (CADD) tool, which allowed potentially deleterious variants from intronic regions of genes to be incorporated into analyses. This work was undertaken using whole-genome sequencing data from the UK10K project. Rare variants from the KEGG pathway for arginine and proline metabolism were collectively associated with systolic blood pressure (*P*=3.32x10^−5^) based on analyses using the optimal sequence kernel association test. Variants along this pathway also showed evidence of replication using imputed data from the Avon Longitudinal Study of Parents and Children cohort (*P=*0.02). Subsequent analyses found that the strength of evidence diminished when analysing genes in this pathway individually, suggesting that they would have been overlooked in a conventional gene-based analysis. Future studies that adopt similar approaches to investigate polygenic effects should yield value in better understanding the genetic architecture of complex disease.

## Introduction

Genome-wide association studies (GWAS) have been successful in identifying novel susceptibility loci across a range of complex diseases. However, the underlying mechanisms by which associated variants influence disease or quantitative phenotypes are often undefined and the variance that they explain is almost always small. Next-generation sequencing (NGS) has allowed rarer genetic variants (that is, minor allele frequency (MAF)<1%), which may potentially contribute substantively to phenotypic variance,^1, 2, 3, 4^ to be genotyped and imputed in larger samples than before. The analysis of these variants, potentially with larger effect sizes,^1^ may prove vital in understanding this missing heritability and uncovering the genetic architecture of complex disease.

Study designs over the last decade have largely concerned the genetic contribution of common variants and thus lack power when applied to rare variants. In order to improve power when analysing these variants using NGS data, methodology based on their aggregation has been developed. Some of these techniques are based on dispersion (c-alpha,^5^ SKAT^6^) and are more robust when analysing rare variants that have conflicting directions of effect for a phenotype of interest (protective or deleterious). Despite this, rare variant analyses remain underpowered, owing to inadequate samples sizes and the manner in which the genome is partitioned. One way in which statistical power can potentially be increased is by analysing combinations of variants which have a similar functional impact from within genes that lie along the same molecular pathway.

Such an alternative approach is attractive for several reasons, including a reduction on the burden for multiple testing and identification of association signals contributed to by multiple genes, which would have otherwise been overlooked using single gene approaches. Expanding the region of interest to collapse variants together across multiple genes also has its challenges, such as choice of pathway definitions. Clearly, aggregating variants together from genes which are not functionally relevant will hinder analyses rather than enrich them. Several studies have already attempted to analyse NGS data with pathway or gene network-based approaches, although generally their findings have been underwhelming.^7, 8, 9^ However, the prospect for developing an approach that successfully identifies polygenic effects from rare variants is potentially very rewarding.

This study aimed to undertake a novel approach to rare variant analysis by focusing on their aggregation across predicted biological pathways, rather than individual loci. Hypothetically, analysing variants from within collective regions may provide stronger evidence of association with phenotypic outcome than single gene approaches. Furthermore, we wanted findings to allow biologically meaningful inferences to be made with regards to the mechanisms of complex disease, which would not be possible when analysing individual genes in isolation. Follow-up analyses of signals detected at a pathway level were performed in order to isolate the evidence of association back to individual genes and variants. We undertook this analysis using whole-genome sequencing (WGS) from participants involved in the UK10K project and cardiovascular phenotypes as measured in the Avon Longitudinal Study of Parents and Children (ALSPAC)^10^ and TwinsUK^11^ cohorts.

## Materials and methods

### Cohort description

The UK10K consortium has two main project arms. In this study, we have used data from the cohorts’ arm, which was designed to investigate the contribution of genome-wide genetic variation to a range of quantitative traits. This arm contains individuals from two intensively studied cohorts of European ancestry, ALSPAC and TwinsUK:

#### ALSPAC

ALSPAC is a population-based cohort study investigating genetic and environmental factors that affect the health and development of children. The study methods are described in detail elsewhere^10, 12^ (http://www.bristol.ac.uk/alspac).

Ethical approval was obtained from the National Research Ethics Service (NRES) Committee, South East London, REC 2. Written informed consent was obtained from parents for all measurements made.

#### TwinsUK

The TwinsUK registry is a cohort of volunteer adult twins from all over the United Kingdom.^11^ Initially, only middle-aged women were recruited and as a result 83% of the registry is female. The registry currently contains 51% monozygotic and 49% dizygotic twins aged 18–103 years. Further details are available online (http://www.twinsuk.ac.uk/).

Informed consent was obtained from participants before they entered the study and ethical approval was granted by the NRES Committee, Westminster, London.

### Sequencing data

DNA Samples from 4030 UK10K study participants (2040 offspring from the ALSPAC cohort, 1990 from the TwinsUK cohort) were subjected to low coverage (6–8 × average read depth) WGS. Sequencing was performed at both the WTSI and the Beijing Genomics Institute (BGI). DNA (1–3 *μ*g) was sheared to 100–1000 bp using a Covaris E210 or LE220 (Covaris, Woburn, MA, USA). Sheared DNA was size subjected to Illumina paired-end DNA library preparation. Following size selection (300–500 bp insert size), DNA libraries were sequenced using the Illumina HiSeq platform as paired-end 100 base reads according to manufacturer’s protocol.

Data that passed quality control (QC) was aligned to the GRCh37 human reference used in phase 1 of the 1000 Genomes Project. Reads were aligned using BWA (v0.5.9-r16).^13^ Of the 4030 participants, 3910 samples (1976 ALSPAC and 1934 TwinsUK) went through the variant calling procedure. Low-quality samples were identified by comparing the samples to their GWAS genotypes using ~20 000 sites on chromosome 20. A total of 112 samples (48 ALSPAC and 64 TwinsUK) were removed, leaving 3798 samples (1928 ALSPAC and 1870 TwinsUK) that were eligible for the genotype refinement phase.

Missing and low-confidence genotypes in the filtered VCFs were refined out using the imputation procedure in BEAGLE 4^14^ with default parameters. Additional sample-level QC steps were carried out on refined genotypes, resulting in 17 samples (16 TwinsUK and 1 ALSPAC) being removed owing to either non-reference discordance with GWAS SNV data>5%, multiple relations to other samples or failed sex check. A principal components analysis was conducted using EIGENSTRAT^15^ to exclude participants of non-European ancestry after merging our data with a pruned 11 HapMap3 population data set.^16^ Forty-four subjects (12 TwinsUK and 32 ALSPAC) did not cluster to the European cluster and were removed. The final sample size for association analyses comprised of 3621 individuals (1754 TwinsUK and 1867 ALSPAC).

### UK10K phenotypes

#### ALSPAC

Body mass index (BMI), systolic blood pressure (SBP), diastolic blood pressure (DBP) and non-fasting blood samples were obtained from the age 9 clinic (mean age: 9.9, range: 8.9–11.5). The blood samples were used to determine lipids including total cholesterol (TC), triglycerides (TG) and high-density lipoproteins (HDL) using a modification of the standard Lipid Research Clinic Protocol.^17^ Low-density lipoproteins were subsequently calculated using the Friedwald equation:^18^

#### TwinsUK

BMI, SBP, DBP and blood samples taken after at least 6 h of fasting were obtained from study participants (mean age: 56, range: 17–85). A colorimetric enzymatic method was used to determine TC, TG and HDL-c levels. The Friedewald equation was used to calculate LDL-c levels in subjects.

Further details on phenotype measurements in the UK10K consortium are provided in the Supplementary Material (Supplementary Information 1).

### ALSPAC imputed data for replication analyses

Genome-wide association (GWA) data was also available for 6557 ALSPAC samples who were not enroled in the UK10K project. This data was genotyped using the Illumina HumanHap550 quad genome-wide SNP genotyping platform (Illumina Inc, San Diego, CA, USA) by the Wellcome Trust Sanger Institute (WTSI, Cambridge, UK) and the Laboratory Corporation of America (LCA, Burlington, NC, USA). All individuals passed QC based on incorrect sex assignment; abnormal heterozygosity (<0.320 or >0.345 for WTSI data; <0.310 or >0.330 for LCA data); high missingness (>3%); cryptic relatedness (>10% identity by descent) and non-European ancestry (detected by multidimensional scaling analysis).

Variants discovered through WGS in the UK10K project were used for the development and use of a reference panel for this imputation within the ALSPAC and TwinsUK GWA data sets. These were combined with known variants taken from the 1000 Genomes reference panel. Novel functionality was developed in IMPUTE2^19, 20^ to use each reference panel to impute missing variants in their counterparts before ultimately combining them together. This meant that all loci involved in the discovery analysis were imputed in this sample. Further detail on this imputation process can be found in the publication by Timpson *et al.*^20^ All rare variants (MAF≤1%) were filtered to have Hardy–Weinberg equilibrium *P*>5 × 10^−7^. As accurate imputation of rare variants can be challenging, only variants with an imputation quality score of 0.4 or higher were used in this analysis. Phenotype measurements were collected as before for lipid traits, although for BMI, SBP and DBP we used measurements obtained from the focus at age 7 clinic (mean age: 7.5, range: 7.1–8.8) in order to maximise sample size.

Please note that the study website contains details of all the data that are available through a fully searchable data dictionary (http://www.bris.ac.uk/alspac/researchers/data-access/data-dictionary). Ethical approval was obtained from the ALSPAC Law and Ethics Committee and the Southmead, Frenchay, UBHT and Weston Research Ethics Committees. Written informed consent was obtained from parents for all measurements made.

### Statistical analysis

#### BioCarta, KEGG and Reactome pathways analyses with cardiovascular traits

Pathway definitions were obtained from three pathway databases (BioCarta,^21^ KEGG^22^ and Reactome^23^). This was achieved by downloading gene sets from the Broad institute’s molecular signatures database (MSigDB, found at http://www.broadinstitute.org/gsea/downloads.jsp) which is used by the GSEA algorithm.^24^ All rare genetic variants (MAF≤1%) that survived QC were aggregated together across pathways according to UCSC reference genome hg19 definitions. We then filtered these sets to only include nonsynonymous variants according to the Ensembl Variant Effect Predictor (VEP).^25^ This process was repeated for a separate analysis except using variants resulting in a loss-of-function (that is, stop gains/losses, splice sites or frameshift indels according to VEP annotations). In a final set of analyses, we used the Combined Annotation Dependent Depletion (CADD)^26^ resource to filter variants based on molecular functionality and pathogenicity. Based on simulations using network analyses surrounding candidate loci (Supplementary Information 2 and Supplementary Figure 1), we hypothesised that a strict threshold would be necessary for this analysis and therefore only included the top 1% most deleterious variants in the UK10K WGS data as predicted by CADD (that is, all variants with a CADD C-Score ≥20).

Rare variants (MAF≤1%) from these regions were then analysed with each cardiovascular trait using the optimal sequence kernel association test (SKAT-O).^27^ Adjustment for the first 10 ancestry principal components was undertaken for all analyses as it has been reported that only adjusting for a small number of top principal components when analysing rare variants can lead to inflated type 1 error rates.^28^ Screeplots suggested that this should be sufficient to account for any potential population stratification in the planned analyses (Supplementary Figure 2).

#### Multiple comparisons and permutation testing

Results that survived the conservative multiple testing threshold (0.05/# of pathways analysed) were further evaluated by performing 100 000 permutation tests. For each test, the same set of genotype values was reanalysed as before except after randomly shuffling phenotype values among individuals. *P*-values were evaluated as robust to permutation if no more than 10 permuted *P*-values were smaller than the *P*-values observed when phenotypes were not shuffled (that is, the observed association).

#### Follow-up analyses to isolate association signals

We followed up pathways that were robust to permutation testing by analysing all genes individually across the genome using the appropriate variant filtering technique and corresponding phenotype. Results were plotted using Manhattan plots, highlighting genes located along the pathway of interest.

We also examined protein–protein interactions to elucidate subnetworks of genes within any pathways with strong evidence of association in the previous analysis. These subnetworks were determined due to the most connected genes in the pathway based on STRINGdb experimental data. Circos plots^29^ were used to visualise how these subnetworks interacted across the genome. Subnetworks of genes were analysed in a similar manner to before in order to isolate association signals across pathways.

#### Replication

Using the imputed ALSPAC data set, we attempted to replicate evidence of association for pathways that survived the multiple testing threshold and were robust to permutation testing. All SNVs in pathways found to be associated in the UK10K data (with imputation quality≥0.4) were collapsed together as before and SKAT-O analyses were repeated using the respective trait. SNVs with an imputation quality score less than this were not included in this analysis. All SNVs in this analysis were also collapsed according to individual gene coordinates to see if there was any overlap with key genes identified in the discovery analysis. As before, analyses were adjusted for the top 10 principal components. Single variant analyses were also undertaken using PLINKv1.9^30^ to verify whether there were any key SNVs in both sets of analyses. All other statistical analyses in this study were undertaken using R statistical software.^31^

## Results

**Figure 1:**
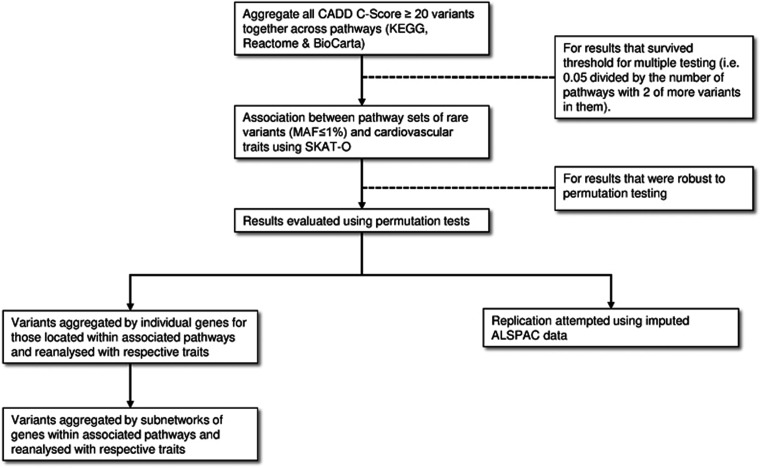
A flowchart illustrating the pipeline for analysis in this study. CADD, combined annotation dependent depletion tool; SKAT-O, optimal sequence kernel association tool; ALSPAC, avon longitudinal study of parents and children.

Figure 1 shows a flowchart of the planned analysis for this study. In total, 3621 individuals with WGS data from the UK10K cohort were available for analysis. After merging individuals with each cardiovascular trait that we planned to analyse, final sample sizes ranged between 3538 and 3191 (3538 for BMI, 3309 for SBP and DBP, 3210 for HDL, 3191 for LDL, 3206 for TC and 3202 for TG).

### KEGG, Reactome and BioCarta pathways analyses with cardiovascular traits

In total, 1095 pathway definitions were obtained from the three pathway databases. We observed no strong evidence of association for any pathway results after filtering to only include nonsynonymous variants based on the multiple testing correction threshold (0.05/1095=*P<*4.57 × 10^−5^). Results can be located in Supplementary Table 1. There were four results that survived the multiple testing correction threshold in the loss-of-function analysis with TG (0.05/897=*P<*5.57 × 10^−5^). However, all these signals were driven by a single rare variant located within the *APOC3* gene region (rs138326449), which has been previously identified in the UK10K data set.^20^ These results can be located in Supplementary Table 2.

The following results for our study now solely concern the analysis based on filtering variants based on their CADD score. One pathway result survived the conservative correction for multiple testing (*P<*4.65 × 10^−5^, that is, 0.05/1.075 based on the number of pathways with two or more rare variants after filtering by a CADD C-Score threshold of ≥20), which was associated with SBP. This was the KEGG arginine and proline Metabolism pathway (*P=*3.32 × 10^−5^), referred to hereafter as the arginine and proline pathway. The top results from this analysis can be located in Table 1. The strongest evidence of association in the BMI analysis was with variants along the *IGF1* pathway according to BioCarta, although this did not survive the threshold for multiple testing (*P=*2.88 × 10^−4^). The top results for this analysis and all other traits can be found in Supplementary Tables 3.

### Permutation testing

No permuted *P*-value survived the multiple testing threshold based on the 100 000 iterations undertaken. Furthermore, only three permuted *P*-values were lower than the observed association between the arginine and proline pathway and SBP (Supplementary Table 4). These results supported evidence that this effect was not due to chance.

### Follow-up analyses to isolate association signals

**Figure 2:**
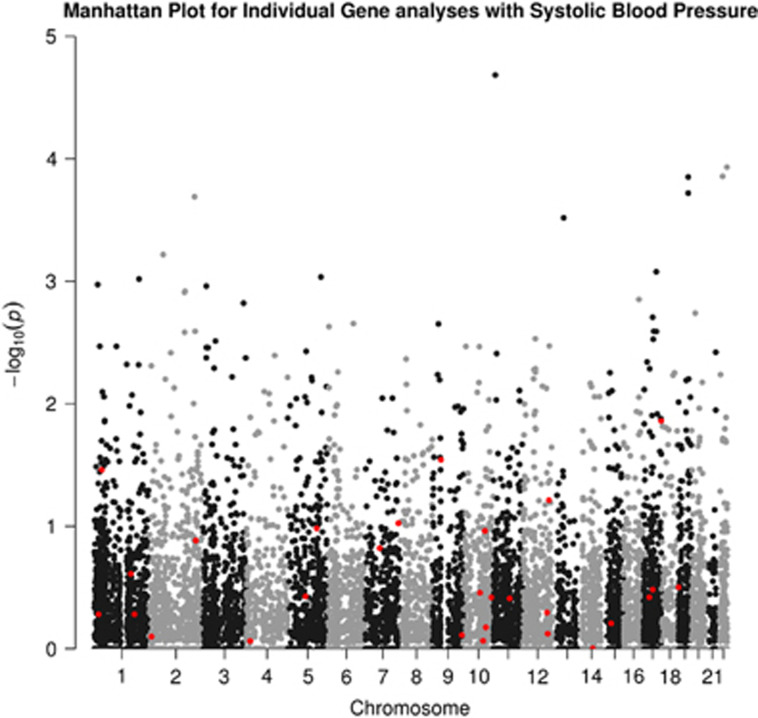
A Manhattan plot illustrating gene-based collapsing of rare variants (MAF≤1%) genome-wide using SKAT-O with systolic blood pressure. Red annotated points represent genes which reside along the arginine and proline pathway.

We collapsed all rare variants (MAF≤1%) across the genome with a CADD C-Score threshold of ≥20 within individual gene regions according to coordinates based on UCSC reference genome hg19 definitions. Each gene-based set of variants was analysed using SKAT-O with SBP. Figure 2 shows a Manhattan plot for the results of this analysis. Genes located along the arginine and proline pathway are highlighted. Notably, none of these *P*-values survived the threshold for multiple comparisons (*P<*6.05 × 10^−6^ (0.05/8269, based on the 8269 gene regions with two or more variants analysed)). This shows that a conventional gene-based approach would not have been able to detect the effect across rare variants in the arginine and proline pathway. Supplementary Table 5 show the results for this individual gene analysis for genes located on this pathway.

**Figure 3:**
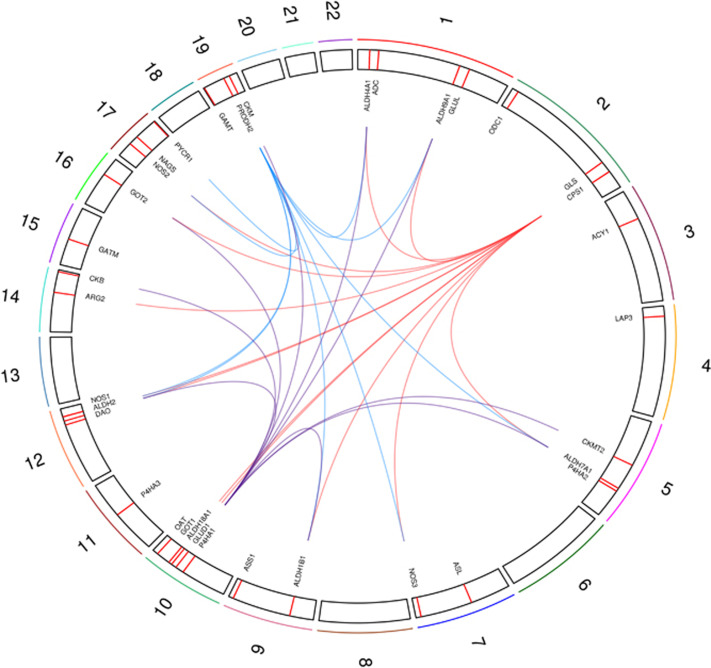
Circos plot representing the genomic location of Genes in the Arginine and Proline pathway and how their protein products interact according to Stringdb. Stringdb suggested that the protein products of three genes along the Arginine and Proline pathway had the most interactions based on experimental evidence. The following three subnetworks are based on the interactions for each of these genes (*CPS1, PRODH2* and *GLUD1*): *CPS1, NOS1, NOS2, NOS3, ALDH9A1, ARG2, ALDH18A1, ALDH1B1, ALDH2, ALDH7A1, ALDH4A1, GOT1* and *GOT2. PRODH2, NOS1, NOS2, NOS3, PYCR1, ALDH9A1, ALDH4A1, ALDH2, ALDH7A1* and *ALDH1B1. GLUD1, CKM, CKMT2, CKB, ALDH1B1, ALDH7A1, ALDH2, ALDH9A1, ALDH4A1* and *GOT2.* Red, blue and purple links are used to differentiate the three subnetworks.

We also examined protein–protein interactions according to Stringdb^32^ experimental data to identify subnetworks of genes within the arginine and proline pathway. There were three genes within this pathway (*CPS1, PRODH2* and *GLUD1*), which had more interactions than any others and therefore subnetworks were based around all genes directly connected to them. Figure 3 displays a Circos plot,^29^ which illustrates interactions between subnetworks within this pathway. Variants within these subnetworks were analysed as before using SKAT-O with SBP. Two subnetworks (the *CPS1* and *GLUD1* subnetworks) provided stronger evidence of association over any individual gene analysis (*P=*1.58 × 10^−4^ and 1.30 × 10^−3^, respectively), however only when all genes in the network were analysed together did evidence become strong enough to survive the threshold for multiple testing in the initial analysis (*P=*4.65 × 10^−5^). Table 2 shows the full results of this analysis.

### Replication using ALSPAC imputed data

We undertook a replication analysis using imputed data from the ALSPAC data set using individuals who were not enroled in the UK10K project (that is, data from a completely separate population of individuals than those already analysed). We replicated evidence of association in the imputed data set between variants along the arginine and proline pathway and SBP (*P=*0.02 from 71 variants, *N=*4380). Evidence of association appeared to be consistent from both the discovery and replication analyses for variants within the *NOS1* gene (*P=*0.06 and *P=*0.05, respectively), although there was little concordance between key individual variant results between the discovery and replication sets of analyses (Supplementary Tables 6–8).

## Discussion

We present an approach to rare variant analysis that expands the region of interest from individual genes to established biological pathways. This approach uses a variant filtering method that incorporates functional annotations from the CADD tool, which allows both intronic and exonic variants to be included into analyses. This is in contrast to conventional filtering methods in rare variant analysis, which are based on variant consequence and are therefore confined to coding regions. Implementing this approach has identified evidence to suggest a set of functionally relevant rare variants collectively influence SBP. Evaluations of this pathway identified subsets of genes that were predominantly responsible for the observed signal, although only when analysed as an entire pathway did statistical evidence become robust enough to survive the correction for multiple comparisons.

The observed association between genes involved in the metabolism of arginine and proline and SBP (*P=*3.32 × 10^−5^ from 115 variants, *N=*3309) in the UK10K data set appeared to replicate in the imputed ALSPAC data set (*P=*0.02 from 71 variants, *N=*4380). There is evidence to suggest that both these amino acids can influence blood pressure, although arginine in particular has gained interest with several studies reporting a blood pressure lowering effect due to arginine dietary supplements.^33, 34, 35^ An important function of arginine is a necessary precursor for the synthesis of nitric oxide, where the impairment of nitric oxide bioavailability has been shown to be a risk factor for hypertension.^36^ *NOS1* was observed to be one of the key genes in both the discovery and replication analyses within this study. This gene belongs to the family of nitric oxide synthases and is involved in the synthesis of nitric oxide from arginine, making it an interesting candidate to explore the mechanisms within this pathway and its effect on blood pressure.

We observed a lack of concordance between key individual variants in the discovery and replication analyses, although this can most likely be explained by the typically observed lack of power when conducting single variant analyses on rare variants.^37, 38^ Replication in another cohort was also contemplated but not undertaken owing to the challenge of identifying a cohort where a sufficient proportion of variants within the discovery analysis were genotyped. Moreover, variants at this frequency are more likely to be population specific in comparison with replication for GWAS hits, which makes replication of rare variant signals challenging, particularly in a trans-ethnic context.^39^ An attractive alternative therefore to the manner in which rare variants have been analysed in this study is to meta-analyse results using software such as RAREMETAL.^40^

As larger sample sizes and lower coverage for sequence data become available this approach should also provide a powerful approach to uncover the role of extremely rare genetic variants (MAF≤0.5%). For future applications of this approach we advise implementing a strict variant filtering phase before analysis in order to remove as much statistical noise as possible. In our initial analysis when filtering variants by conventional methods based on variant consequence we did not observe any novel evidence of association in the UK10K data set. However, when incorporating annotations from CADD we were able to identify a previously undetected signal. Although other studies have incorporated non-coding annotations into their analyses to great effect,^41^ this study presents an approach for utilising them in the context of rare variant analysis within a pathway-centric framework. This is a noteworthy point as when performing a conventional gene-based analysis evidence of association for this novel signal diminished.

The limitation of this approach is that potential causal variants may be filtered out when based on CADD predictions. In this study we have chosen pathway definitions using the KEGG, Reactome and BioCarta resources, although future studies may benefit from incorporating gene sets based on alternative definitions. We have also showcased SKAT-O as an adaptive method suited to this approach. Along with its capability to identify phenotype associated regions of variation, which can have conflicting directions of effect, the manner in which SKAT-O adjusts for LD, using the Davies method,^42^ also makes it an attractive approach when analysing multiple genes in a pathway which can potentially have close proximity. As reported by other studies, analysing pathway sets of variants using SKAT-O, or with any other collapsing method, remains relatively underpowered.^7, 8^ Power calculations from the replication analysis in this study also support this, as evaluations found observed power to diminish as the sample size and percentage of variants in the sample were reduced (Supplementary Information 3).

Success from rare variant association analyses has been limited thus far.^43, 44^ In recent years, studies have attempted to improve statistical power in their approaches by increasing their sample sizes,^45^ adapting current methodological techniques^46^ or conducting analyses on remote or isolated populations where the allele frequency of rarer variants may be heightened.^47^ By expanding our definition of a biologically functional unit from individual genes, we have provided an alternative method to leverage statistical power. Furthermore, subsequent analysis concerning pathway enrichment (for example, GSEA^24^) would struggle to detect an over representation of genes along certain pathways. Our approach allows pathological mechanistic insights to be made which methods involving individual gene regions would not. This is also an advantage over individual variant methods, such as GWAS, where the majority of robustly associated findings lie in non-coding regions, leaving questions regarding the variants’ functional impact.^48^ As larger samples of low coverage sequence data become available, future studies should benefit from using this approach to uncover the genetic architecture of complex traits that contribute to disease.

## Figures and Tables

**Table 1:** Systolic blood pressure results using a minor allele frequency cutoff of 1%

*Pathway*	*Variants*^a^	*UK10K* P*-value*	*TwinsUK* P*-value*	*ALSPAC* P*-value*
KEGG: arginine and proline metabolism	115	3.32 × 10^−5^	7.42 × 10^−4^	0.04
Reactome: amino acid synthesis and interconversion transamination	26	9.69 × 10^−4^	0.04	0.06
KEGG: antigen processing and presentation	18	5.78 × 10^−3^	0.27	0.02
Reactome: synthesis secretion and deacylation of ghrelin	13	6.12 × 10^−3^	0.04	0.13
KEGG: tight junction	412	8.48 × 10^−3^	0.29	0.36
KEGG: Jak Stat signalling pathway	90	0.01	0.34	0.04
BioCarta: cytokine pathway	5	0.01	1.91 × 10^−3^	0.48
Reactome: regulation of IFNG signalling	20	0.01	0.38	0.02
Reactome: assembly of the pre replicative comple ×	97	0.02	0.12	0.12
BioCarta: RAB pathway	9	0.02	0.15	0.05

a Variants=number of variants, UK10K *P*-value=SKAT-O *P*-value for entire UK10K sample, Twins *P*-value, SKAT-O *P*-value for only TwinsUK individuals, ALSPAC *P*-value=SKAT-O *P*-value for only ALSPAC individuals.

**Table 2:** Subnetwork analysis for the KEGG arginine and proline metabolism pathway with systolic blood pressure

	*UK10K*		*TwinsUK*		*ALSPAC*	
*Genes in Subnetwork*	*Variants*^a^	P*-value*	*Variants*^a^	P*-value*	*Variants*^a^	P*-value*
*CPS1, NOS1, NOS2, NOS3, ALDH9A1, ARG2, ALDH18A1, ALDH1B1, ALDH2, ALDH7A1, ALDH4A1, GOT1* and *GOT2*	57	1.58 × 10^−4^	38	4.00 × 10^−3^	40	0.03
*PRODH2, NOS1, NOS2, NOS3, PYCR1, ALDH9A1, ALDH4A1, ALDH2, ALDH7A1* and *ALDH1B1*	25	0.02	18	0.16	20	0.15
*GLUD1, CKM, CKMT2, CKB, ALDH1B1, ALDH7A1, ALDH2, ALDH9A1, ALDH4A1* and *GOT2*	34	1.30 × 10^−3^	25	0.02	26	0.06

a Variants=number of variants analysed, UK10K=all individuals within UK10K project, TwinsUK=only TwinsUK partition of the UK10K project, ALSPAC=only ALSPAC partition of the UK10K project, *P*-value=*P*-values according to SKAT-O test.
