# Supplementary material for: A pathway-centric approach to rare variant association analysis
Source: Eur J Hum Genet. 2016 Aug 31;25(1):123–9. doi: 10.1038/ejhg.2016.113 (PMC5136291; doi:10.1038/ejhg.2016.113)
Supplement: Supplementary Information [file ejhg2016113x1.doc]

**Supplementary Material**

## **Supplementary Information 1: UK10K Phenotypes**

*ALSPAC:* Height was measured to the nearest 0.1cm using a Harpenden stadiometer (Holtain Crosswell, Dyfed, UK) and weight was measured to the nearest 0.1kg using Tanita electronic scales. Body Mass Index (BMI) was calculated as (weight (kg))/(height (m))^2^. Blood Pressure was measured with a Dinamap 9301 vital monitor completed by trained staff using the appropriate cuff size. Two readings of both systolic and diastolic blood pressure (SBP & DBP respectively) were taken when the study participants were at rest and the mean of each were used as a measurement in our analysis. Both these measurements were taken from the age 9 clinic (mean age: 9.9, range: 8.9–11.5).

Non-fasting blood samples were also taken from participants who attended the age 9 clinic (mean age: 9.9, range: 8.9 – 11.5). Plasma lipid concentrations (total cholesterol (TC), triglycerides (TG) and high density lipoprotein cholesterol (HDLc)) were measured by modification of the standard Lipid Research Clinics Protocol with enzymatic reagents for lipid determination^19^. Low density lipoprotein cholesterol (LDLc) concentration was subsequently calculated using the Friedwald equation^20^:

*LDLc = TC – (HDLc + TG×0.45)*

*TwinsUK:* Height was measured to the nearest 0.5cm using a wall-mounted stadiometer and weight (light clothing only) was measured to the nearest 0.1kg using digital scales. Body Mass Index (BMI) was calculated as (weight (kg))/(height (m))^2^. Brachial blood pressure was measured using an automated cuff sphygmomanometer (OMRON HEM713C; Omron Healthcare (UK) Ltd, Henfield, UK). SBP and DBP were measured three times, two of which were highly correlated (0.90 for SBP and 0.92 for DBP) and averaged to get our final phenotype measurements.

Blood samples were taken after at least 6 hours of overnight fasting. The samples were immediately inverted three times and left to rest for 40 minutes at 4ºC to obtain complete coagulation. The samples were then centrifuged for 10 min at 2000g and serum was removed. Four aliquots of 1.5 ml were placed into skirted micro centrifuge tubes and then stored in a -45ºC freezer until sampling^21^. A colorimetric enzymatic method was used to determine TC, TG and HDL-c levels. The Friedewald equation was used to calculate LDL-c levels in subjects.

## **Supplementary Information 2: Simulated Pathway Analysis around Candidate loci**

Using the HDL data from the UK10K project cohort arm, we examined how evidence of association from rare variants (MAF≤1%) within candidate genes held up when analysed as part of networks of varying length. Firstly, we filtered our sequence data to only include variants that were ‘nonsynonymous’ according to dbSNP annotations (build 137). We identified *CETP* as our candidate gene which has previously shown robust evidence of being associated with HDL levels^17; 18^. We subsequently generated gene networks of varying length whose products had strong experimental evidence of interaction according to the String v9.1 database (STRINGdb)^19^ and randomly replaced one of these genes with *CETP*. We then took all variants across all genes in each network, according to UCSC reference genome hg19 definitions, and analysed then together with HDL using SKAT. We repeated this process 10,000 times for each length of network (2, 3, 5, 10, 15, 20, 25 and 30 genes).

We repeated this process using *CETP* and its neighbouring gene *LPL* as our candidate genes. Finally, we repeated this process with the triplet of interacting genes *CETP*, *LPL* and *APOE.* We took the average p-value from each of the 10,000 simulations using each length of network and plotted these results together to examine how different strengths of association signal held up when observed within varying lengths of gene network.

## **Supplementary Figure 1: Variation in Evidence of Signal across Network from known candidate loci**


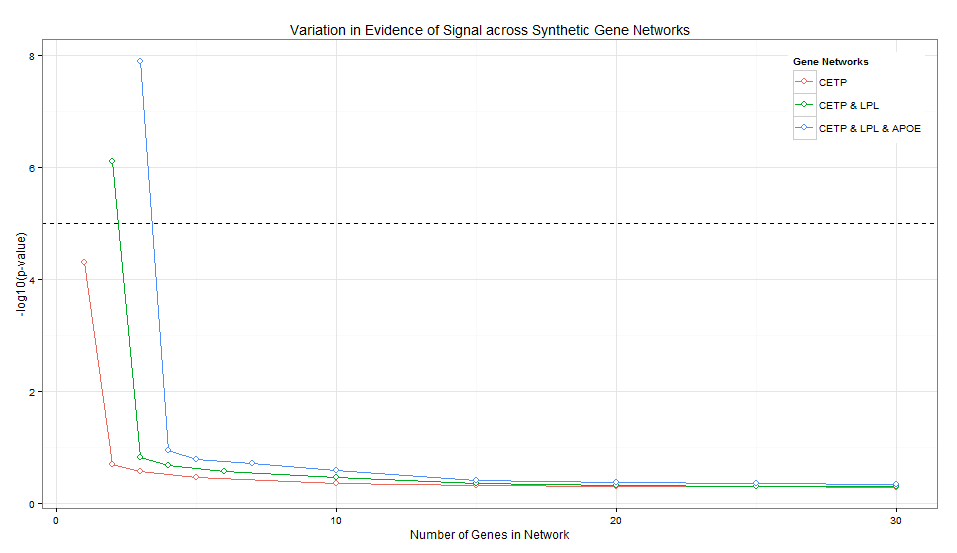


## **Supplementary Figure 2: Screeplots**

1. Screeplot for genetic data after Nonsynonymous filtering


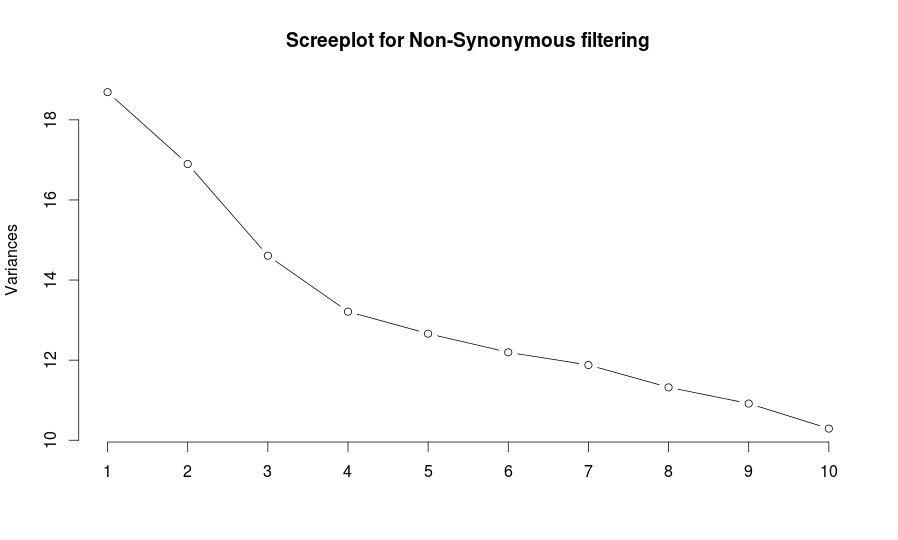


1. Screeplot for genetic data after Loss-of-Function filtering


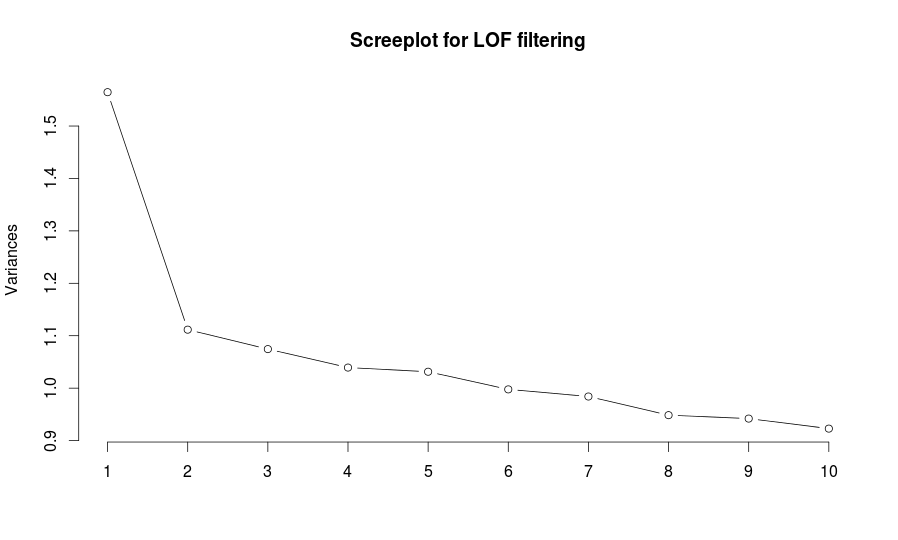


1. Screeplot for genetic data after CADD filtering


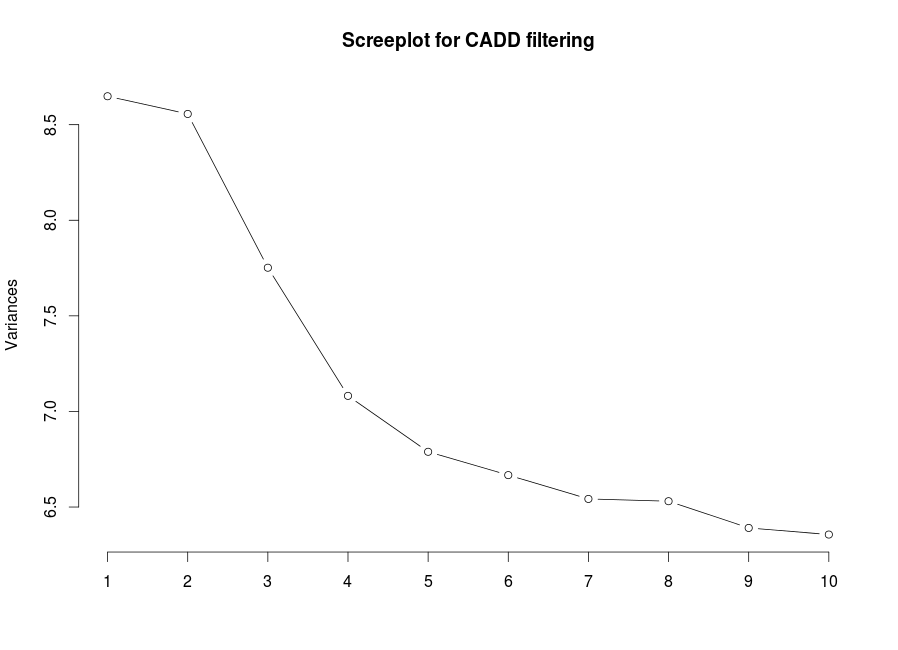


## **Supplementary Tables 1: SKAT-O analyses for Nonsynonymous variants using a MAF cutoff of 1%**

Supplementary table 1a BMI:

| **Pathway** | **# variants** | **P-value** |
| --- | --- | --- |
| REACTOME_RNA_POL_I_PROMOTER_OPENING | 50 | 0.005378 |
| REACTOME_GAP_JUNCTION_TRAFFICKING | 146 | 0.008125 |
| REACTOME_TRAF6_MEDIATED_IRF7_ACTIVATION_IN_TLR7_8_OR_9_SIGNALING | 27 | 0.009092 |
| REACTOME_MEIOTIC_SYNAPSIS | 416 | 0.012941 |
| BIOCARTA_CARM_ER_PATHWAY | 272 | 0.017705 |
| BIOCARTA_ARF_PATHWAY | 86 | 0.017762 |
| REACTOME_INSULIN_RECEPTOR_RECYCLING | 113 | 0.021559 |
| REACTOME_LIGAND_GATED_ION_CHANNEL_TRANSPORT | 63 | 0.023676 |
| BIOCARTA_IL10_PATHWAY | 80 | 0.028289 |
| REACTOME_GPCR_LIGAND_BINDING | 133 | 0.030008 |

Supplementary table 1b SBP:

| **Pathway** | **# variants** | **P-value** |
| --- | --- | --- |
| KEGG_RENAL_CELL_CARCINOMA | 270 | 0.00118 |
| KEGG_COLORECTAL_CANCER | 325 | 0.001307 |
| KEGG_GRAFT_VERSUS_HOST_DISEASE | 76 | 0.00323 |
| REACTOME_ENDOSOMAL_VACUOLAR_PATHWAY | 11 | 0.004162 |
| KEGG_ALLOGRAFT_REJECTION | 70 | 0.004166 |
| KEGG_ANTIGEN_PROCESSING_AND_PRESENTATION | 74 | 0.004566 |
| KEGG_TRYPTOPHAN_METABOLISM | 220 | 0.006363 |
| KEGG_GLIOMA | 300 | 0.007032 |
| BIOCARTA_CALCINEURIN_PATHWAY | 81 | 0.008457 |
| REACTOME_SULFUR_AMINO_ACID_METABOLISM | 109 | 0.009118 |

Supplementary table 1c DBP:

| **Pathway** | **# variants** | **P-value** |
| --- | --- | --- |
| BIOCARTA_GLEEVEC_PATHWAY | 84 | 0.00236 |
| BIOCARTA_BCELLSURVIVAL_PATHWAY | 66 | 0.004127 |
| REACTOME_NUCLEOTIDE_BINDING_DOMAIN_LEUCINE_RICH_REPEAT | 259 | 0.004462 |
| BIOCARTA_UCALPAIN_PATHWAY | 119 | 0.00543 |
| KEGG_TRYPTOPHAN_METABOLISM | 224 | 0.007368 |
| REACTOME_AMINE_DERIVED_HORMONES | 95 | 0.008638 |
| KEGG_MTOR_SIGNALING_PATHWAY | 250 | 0.00878 |
| REACTOME_ACTIVATED_TAK1_MEDIATES_P38_MAPK_ACTIVATION | 85 | 0.010201 |
| KEGG_CELL_ADHESION_MOLECULES_CAMS | 252 | 0.012148 |
| REACTOME_SULFUR_AMINO_ACID_METABOLISM | 111 | 0.014048 |

Supplementary table 1d HDL:

| **Pathway** | **# variants** | **P-value** |
| --- | --- | --- |
| REACTOME_POST_TRANSLATIONAL_MODIFICATION_SYNTHESIS_OF_GPI_ANCHORED_PROTEINS | 163 | 0.002387 |
| REACTOME_REGULATION_OF_IFNG_SIGNALING | 52 | 0.002997 |
| REACTOME_INHIBITION_OF_THE_PROTEOLYTIC_ACTIVITY_OF_APC | 46 | 0.00394 |
| KEGG_SNARE_INTERACTIONS_IN_VESICULAR_TRANSPORT | 124 | 0.005515 |
| REACTOME_APC_CDC20_MEDIATED_DEGRADATION_OF_NEK2A | 52 | 0.006376 |
| REACTOME_MHC_CLASS_II_ANTIGEN_PRESENTATION | 221 | 0.007039 |
| REACTOME_RNA_POL_III_TRANSCRIPTION_INITIATION_FROM_TYPE_3_PROMOTER | 130 | 0.009212 |
| REACTOME_ACTIVATION_OF_THE_MRNA_UPON_BINDING_OF_THE_CAP_BINDING_COMPLEX | 64 | 0.009613 |
| BIOCARTA_FREE_PATHWAY | 50 | 0.009711 |
| BIOCARTA_ACH_PATHWAY | 68 | 0.010182 |

Supplementary table 1e LDL:

| **Pathway** | **# variants** | **P-value** |
| --- | --- | --- |
| REACTOME_GRB2_EVENTS_IN_ERBB2_SIGNALING | 94 | 0.000861 |
| REACTOME_SYNTHESIS_OF_BILE_ACIDS_AND_BILE_SALTS_VIA_24_HYDROXYCHOLESTEROL | 66 | 0.001698 |
| REACTOME_GABA_B_RECEPTOR_ACTIVATION | 172 | 0.001789 |
| REACTOME_ADENYLATE_CYCLASE_INHIBITORY_PATHWAY | 113 | 0.002771 |
| REACTOME_STEROID_HORMONES | 217 | 0.003497 |
| BIOCARTA_EIF2_PATHWAY | 165 | 0.003526 |
| REACTOME_GABA_RECEPTOR_ACTIVATION | 221 | 0.005651 |
| KEGG_PRIMARY_BILE_ACID_BIOSYNTHESIS | 116 | 0.006128 |
| REACTOME_METABOLISM_OF_STEROID_HORMONES_AND_VITAMINS_A_AND_D | 233 | 0.006587 |
| REACTOME_MICRORNA_MIRNA_BIOGENESIS | 60 | 0.007913 |

Supplementary table 1f TC:

| **Pathway** | **# variants** | **P-value** |
| --- | --- | --- |
| REACTOME_OTHER_SEMAPHORIN_INTERACTIONS | 124 | 0.005227 |
| REACTOME_GABA_B_RECEPTOR_ACTIVATION | 177 | 0.005331 |
| REACTOME_CD28_DEPENDENT_VAV1_PATHWAY | 24 | 0.006828 |
| REACTOME_UNWINDING_OF_DNA | 76 | 0.007778 |
| KEGG_HYPERTROPHIC_CARDIOMYOPATHY_HCM | 1038 | 0.00803 |
| REACTOME_GRB2_EVENTS_IN_ERBB2_SIGNALING | 94 | 0.009346 |
| REACTOME_L1CAM_INTERACTIONS | 538 | 0.010964 |
| KEGG_LEUKOCYTE_TRANSENDOTHELIAL_MIGRATION | 438 | 0.011176 |
| REACTOME_ADENYLATE_CYCLASE_INHIBITORY_PATHWAY | 113 | 0.011544 |
| REACTOME_MICRORNA_MIRNA_BIOGENESIS | 60 | 0.011772 |

Supplementary table 1g TG:

| **Pathway** | **# variants** | **P-value** |
| --- | --- | --- |
| REACTOME_OTHER_SEMAPHORIN_INTERACTIONS | 124 | 0.000399 |
| BIOCARTA_HDAC_PATHWAY | 143 | 0.003321 |
| KEGG_SNARE_INTERACTIONS_IN_VESICULAR_TRANSPORT | 124 | 0.004896 |
| KEGG_PANCREATIC_CANCER | 327 | 0.008693 |
| REACTOME_ANTIVIRAL_MECHANISM_BY_IFN_STIMULATED_GENES | 318 | 0.010214 |
| KEGG_FC_EPSILON_RI_SIGNALING_PATHWAY | 316 | 0.014272 |
| REACTOME_RNA_POL_I_PROMOTER_OPENING | 50 | 0.01556 |
| REACTOME_P38MAPK_EVENTS | 51 | 0.019665 |
| KEGG_COLORECTAL_CANCER | 325 | 0.02066 |
| KEGG_CYSTEINE_AND_METHIONINE_METABOLISM | 162 | 0.02144 |

## **Supplementary Tables 2: SKAT-O analyses for Loss-of-Function variants using a MAF cutoff of 1%**

**Supplementary table 2a BMI**:

| **Pathway** | **# variants** | **P-value** |
| --- | --- | --- |
| REACTOME_NEGATIVE_REGULATORS_OF_RIG_I_MDA5_SIGNALING | 7 | 0.000768 |
| REACTOME_TRAF3_DEPENDENT_IRF_ACTIVATION_PATHWAY | 7 | 0.000799 |
| REACTOME_NFKB_ACTIVATION_THROUGH_FADD_RIP1_PATHWAY_MEDIATED_BY_CASPASE_8_AND10 | 7 | 0.001199 |
| REACTOME_TRAF6_MEDIATED_NFKB_ACTIVATION | 7 | 0.001446 |
| KEGG_CELL_ADHESION_MOLECULES_CAMS | 7 | 0.003254 |
| BIOCARTA_PARKIN_PATHWAY | 4 | 0.004949 |
| REACTOME_ENDOSOMAL_SORTING_COMPLEX_REQUIRED_FOR_TRANSPORT_ESCRT | 3 | 0.005015 |
| REACTOME_TRAF6_MEDIATED_IRF7_ACTIVATION | 9 | 0.006431 |
| REACTOME_SEROTONIN_RECEPTORS | 3 | 0.006595 |
| REACTOME_RIG_I_MDA5_MEDIATED_INDUCTION_OF_IFN_ALPHA_BETA_PATHWAYS | 10 | 0.00686 |

**Supplementary table 2b SBP**:

| **Pathway** | **# variants** | **P-value** |
| --- | --- | --- |
| REACTOME_ACTIVATION_OF_ATR_IN_RESPONSE_TO_REPLICATION_STRESS | 9 | 0.001237 |
| REACTOME_OXYGEN_DEPENDENT_PROLINE_HYDROXYLATION_OF_HYPOXIA_INDUCIBLE_FACTOR_ALPHA | 2 | 0.002336 |
| REACTOME_FATTY_ACYL_COA_BIOSYNTHESIS | 3 | 0.002467 |
| REACTOME_SYNTHESIS_OF_VERY_LONG_CHAIN_FATTY_ACYL_COAS | 3 | 0.002467 |
| REACTOME_STEROID_HORMONES | 6 | 0.003079 |
| REACTOME_REGULATION_OF_HYPOXIA_INDUCIBLE_FACTOR_HIF_BY_OXYGEN | 4 | 0.004506 |
| REACTOME_G2_M_CHECKPOINTS | 11 | 0.004675 |
| BIOCARTA_ATRBRCA_PATHWAY | 14 | 0.005349 |
| BIOCARTA_EIF2_PATHWAY | 3 | 0.005496 |
| REACTOME_METABOLISM_OF_STEROID_HORMONES_AND_VITAMINS_A_AND_D | 8 | 0.007093 |

**Supplementary table 2c DBP**:

| **Pathway** | **# variants** | **P-value** |
| --- | --- | --- |
| REACTOME_G_ALPHA_Q_SIGNALLING_EVENTS | 9 | 0.003622 |
| REACTOME_GASTRIN_CREB_SIGNALLING_PATHWAY_VIA_PKC_AND_MAPK | 10 | 0.003817 |
| REACTOME_ELEVATION_OF_CYTOSOLIC_CA2_LEVELS | 2 | 0.00499 |
| REACTOME_ROLE_OF_SECOND_MESSENGERS_IN_NETRIN1_SIGNALING | 2 | 0.010587 |
| KEGG_CYTOSOLIC_DNA_SENSING_PATHWAY | 22 | 0.011148 |
| REACTOME_EFFECTS_OF_PIP2_HYDROLYSIS | 9 | 0.012433 |
| REACTOME_PLATELET_CALCIUM_HOMEOSTASIS | 5 | 0.012767 |
| REACTOME_TRANSPORT_OF_MATURE_TRANSCRIPT_TO_CYTOPLASM | 6 | 0.023085 |
| REACTOME_INHIBITION_OF_VOLTAGE_GATED_CA2_CHANNELS_VIA_GBETA_GAMMA_SUBUNITS | 2 | 0.025127 |
| REACTOME_REGULATION_OF_GLUCOKINASE_BY_GLUCOKINASE_REGULATORY_PROTEIN | 6 | 0.027839 |

**Supplementary table 2d HDL**:

| **Pathway** | **# variants** | **P-value** |
| --- | --- | --- |
| REACTOME_HDL_MEDIATED_LIPID_TRANSPORT | 6 | 0.000106 |
| REACTOME_CHYLOMICRON_MEDIATED_LIPID_TRANSPORT | 4 | 0.000284 |
| REACTOME_RNA_POL_I_TRANSCRIPTION | 5 | 0.001596 |
| KEGG_PPAR_SIGNALING_PATHWAY | 30 | 0.008422 |
| REACTOME_LIPID_DIGESTION_MOBILIZATION_AND_TRANSPORT | 15 | 0.010009 |
| REACTOME_UNFOLDED_PROTEIN_RESPONSE | 16 | 0.010945 |
| REACTOME_ACTIVATION_OF_GENES_BY_ATF4 | 9 | 0.01698 |
| REACTOME_PERK_REGULATED_GENE_EXPRESSION | 9 | 0.01698 |
| REACTOME_LIPOPROTEIN_METABOLISM | 11 | 0.01709 |
| REACTOME_MEIOSIS | 19 | 0.02309 |

**Supplementary table 2e LDL**:

| **Pathway** | **# variants** | | **P-value** | |
| --- | --- | --- | --- | --- |
| REACTOME_XENOBIOTICS | 5 | 0.00217 | |  |
| REACTOME_CELL_SURFACE_INTERACTIONS_AT_THE_VASCULAR_WALL | 14 | 0.003362 | |  |
| REACTOME_DOUBLE_STRAND_BREAK_REPAIR | 6 | 0.006012 | |  |
| REACTOME_HOMOLOGOUS_RECOMBINATION_REPAIR_OF_REPLICATION | 6 | 0.006012 | |  |
| REACTOME_ANTIGEN_PROCESSING_CROSS_PRESENTATION | 9 | 0.009681 | |  |
| REACTOME_HORMONE_LIGAND_BINDING_RECEPTORS | 3 | 0.010337 | |  |
| KEGG_HOMOLOGOUS_RECOMBINATION | 13 | 0.010376 | |  |
| BIOCARTA_PPARA_PATHWAY | 7 | 0.010995 | |  |
| REACTOME_AUTODEGRADATION_OF_THE_E3_UBIQUITIN_LIGASE_COP1 | 5 | 0.015289 | |  |
| REACTOME_BASIGIN_INTERACTIONS | 9 | 0.015328 | |  |

**Supplementary table 2f TC**:

| **Pathway** | **# variants** | **P-value** |
| --- | --- | --- |
| KEGG_FRUCTOSE_AND_MANNOSE_METABOLISM | 14 | 0.005662 |
| REACTOME_HYALURONAN_UPTAKE_AND_DEGRADATION | 5 | 0.00817 |
| REACTOME_BIOSYNTHESIS_OF_THE_N_GLYCAN_PRECURSOR_DOLICHOL_LIPID | 5 | 0.012768 |
| REACTOME_NEUROTRANSMITTER_RECEPTOR_BINDING | 20 | 0.016119 |
| KEGG_RETINOL_METABOLISM | 25 | 0.017307 |
| KEGG_PORPHYRIN_AND_CHLOROPHYLL_METABOLISM | 15 | 0.017968 |
| REACTOME_METABOLISM_OF_NON_CODING_RNA | 7 | 0.019845 |
| REACTOME_PPARA_ACTIVATES_GENE_EXPRESSION | 15 | 0.022233 |
| KEGG_INTESTINAL_IMMUNE_NETWORK_FOR_IGA_PRODUCTION | 8 | 0.022876 |
| REACTOME_LIPOPROTEIN_METABOLISM | 11 | 0.026919 |

**Supplementary table 2g TG**:

| Pathway | # variants | P-value |
| --- | --- | --- |
| REACTOME_HDL_MEDIATED_LIPID_TRANSPORT | 6 | 3.29E-09 |
| REACTOME_CHYLOMICRON_MEDIATED_LIPID_TRANSPORT | 4 | 6.58E-09 |
| REACTOME_LIPOPROTEIN_METABOLISM | 11 | 6.09E-06 |
| REACTOME_LIPID_DIGESTION_MOBILIZATION_AND_TRANSPORT | 15 | 2.14E-05 |
| REACTOME_APOPTOTIC_CLEAVAGE_OF_CELLULAR_PROTEINS | 8 | 0.001324 |
| REACTOME_APOPTOTIC_EXECUTION_PHASE | 8 | 0.001929 |
| KEGG_PPAR_SIGNALING_PATHWAY | 30 | 0.002231 |
| REACTOME_NEGATIVE_REGULATORS_OF_RIG_I_MDA5_SIGNALING | 7 | 0.003098 |
| REACTOME_NFKB_ACTIVATION_THROUGH_FADD_RIP1_PATHWAY_MEDIATED_BY_CASPASE_8_AND10 | 7 | 0.003117 |
| REACTOME_TRAF3_DEPENDENT_IRF_ACTIVATION_PATHWAY | 7 | 0.003618 |

## **Supplementary Tables 3: SKAT-O analyses for variants with a CADD C-Score ≥ 20 (i.e. the 1% most deleterious variants in the UK10K dataset) using a MAF cutoff of 1%**

**Supplementary Table 3a BMI**

| **Pathway** | **# variants** | **P-value** |
| --- | --- | --- |
| BIOCARTA_IGF1_PATHWAY | 53 | 0.000288 |
| BIOCARTA_IL4_PATHWAY | 14 | 0.000328 |
| REACTOME_GAP_JUNCTION_DEGRADATION | 38 | 0.000863 |
| KEGG_LONG_TERM_DEPRESSION | 283 | 0.000894 |
| REACTOME_G_PROTEIN_BETA_GAMMA_SIGNALLING | 50 | 0.000912 |
| REACTOME_THROMBIN_SIGNALLING_THROUGH_PROTEINASE_ACTIVATED_RECEPTORS_PARS | 36 | 0.000984 |
| BIOCARTA_LONGEVITY_PATHWAY | 48 | 0.000995 |
| KEGG_ALDOSTERONE_REGULATED_SODIUM_REABSORPTION | 102 | 0.001034 |
| BIOCARTA_ERYTH_PATHWAY | 25 | 0.001601 |
| REACTOME_CLASS_B_2_SECRETIN_FAMILY_RECEPTORS | 69 | 0.00175 |

**Supplementary Table 3b** DBP

| **Pathway** | **# variants** | **P-value** |
| --- | --- | --- |
| REACTOME_INTERFERON_ALPHA_BETA_SIGNALING | 29 | 0.002187 |
| BIOCARTA_DEATH_PATHWAY | 67 | 0.002838 |
| BIOCARTA_KERATINOCYTE_PATHWAY | 142 | 0.003338 |
| REACTOME_REGULATION_OF_IFNA_SIGNALING | 13 | 0.004267 |
| BIOCARTA_NFKB_PATHWAY | 26 | 0.007441 |
| REACTOME_DOWNSTREAM_SIGNALING_EVENTS_OF_B_CELL_RECEPTOR_BCR | 139 | 0.00981 |
| BIOCARTA_LEPTIN_PATHWAY | 36 | 0.010121 |
| REACTOME_CELL_CYCLE | 287 | 0.010382 |
| REACTOME_COSTIMULATION_BY_THE_CD28_FAMILY | 77 | 0.010825 |
| REACTOME_G_ALPHA_Z_SIGNALLING_EVENTS | 134 | 0.013394 |

**Supplementary Table 3c** HDL

| **Pathway** | **# variants** | **P-value** |
| --- | --- | --- |
| REACTOME_PLC_BETA_MEDIATED_EVENTS | 154 | 0.000884 |
| REACTOME_RNA_POL_III_CHAIN_ELONGATION | 66 | 0.001268 |
| REACTOME_RNA_POL_III_TRANSCRIPTION_INITIATION_FROM_TYPE_2_PROMOTER | 69 | 0.001331 |
| REACTOME_RNA_POL_I_RNA_POL_III_AND_MITOCHONDRIAL_TRANSCRIPTION | 130 | 0.001571 |
| REACTOME_RNA_POL_III_TRANSCRIPTION_INITIATION_FROM_TYPE_3_PROMOTER | 81 | 0.003536 |
| REACTOME_FORMATION_OF_TRANSCRIPTION_COUPLED_NER_TC_NER_REPAIR_COMPLEX | 68 | 0.003887 |
| REACTOME_OPSINS | 16 | 0.003979 |
| REACTOME_RNA_POL_I_TRANSCRIPTION_INITIATION | 55 | 0.004499 |
| KEGG_APOPTOSIS | 130 | 0.004679 |
| REACTOME_RNA_POL_III_TRANSCRIPTION_TERMINATION | 98 | 0.004712 |

**Supplementary Table 3d** LDL

| **Pathway** | **# variants** | **P-value** |
| --- | --- | --- |
| KEGG_DRUG_METABOLISM_CYTOCHROME_P450 | 80 | 0.00184 |
| KEGG_GLYCOSAMINOGLYCAN_BIOSYNTHESIS_HEPARAN_SULFATE | 151 | 0.002016 |
| REACTOME_XENOBIOTICS | 18 | 0.002045 |
| KEGG_PATHWAYS_IN_CANCER | 651 | 0.002438 |
| REACTOME_NUCLEOTIDE_LIKE_PURINERGIC_RECEPTORS | 9 | 0.005064 |
| REACTOME_GLYCEROPHOSPHOLIPID_BIOSYNTHESIS | 180 | 0.005201 |
| REACTOME_REGULATION_OF_PYRUVATE_DEHYDROGENASE_PDH_COMPLEX | 16 | 0.005273 |
| REACTOME_ROLE_OF_DCC_IN_REGULATING_APOPTOSIS | 87 | 0.005446 |
| KEGG_METABOLISM_OF_XENOBIOTICS_BY_CYTOCHROME_P450 | 73 | 0.006292 |
| KEGG_LINOLEIC_ACID_METABOLISM | 44 | 0.007077 |

**Supplementary Table 3e** TC

| **Pathway** | **# variants** | **P-value** |
| --- | --- | --- |
| REACTOME_P75_NTR_RECEPTOR_MEDIATED_SIGNALLING | 238 | 0.001329 |
| REACTOME_G2_M_CHECKPOINTS | 77 | 0.00143 |
| BIOCARTA_MCM_PATHWAY | 43 | 0.001579 |
| REACTOME_IRAK1_RECRUITS_IKK_COMPLEX | 11 | 0.002293 |
| REACTOME_ACTIVATION_OF_ATR_IN_RESPONSE_TO_REPLICATION_STRESS | 82 | 0.002351 |
| REACTOME_FORMATION_OF_ATP_BY_CHEMIOSMOTIC_COUPLING | 12 | 0.002889 |
| REACTOME_MRNA_CAPPING | 41 | 0.003266 |
| REACTOME_CELL_DEATH_SIGNALLING_VIA_NRAGE_NRIF_AND_NADE | 161 | 0.003293 |
| BIOCARTA_RAC1_PATHWAY | 86 | 0.003552 |
| REACTOME_ACTIVATION_OF_THE_PRE_REPLICATIVE_COMPLEX | 80 | 0.004469 |

**Supplementary Table 3f** TG

| **Pathway** | **# variants** | **P-value** |
| --- | --- | --- |
| KEGG_DRUG_METABOLISM_CYTOCHROME_P450 | 80 | 0.002516 |
| BIOCARTA_AKAP13_PATHWAY | 28 | 0.002628 |
| BIOCARTA_ETC_PATHWAY | 28 | 0.005543 |
| REACTOME_NFKB_ACTIVATION_THROUGH_FADD_RIP1_PATHWAY | 26 | 0.006412 |
| REACTOME_INCRETIN_SYNTHESIS_SECRETION_AND_INACTIVATION | 36 | 0.006624 |
| REACTOME_TRAF6_MEDIATED_NFKB_ACTIVATION | 36 | 0.008108 |
| REACTOME_ZINC_TRANSPORTERS | 21 | 0.011053 |
| REACTOME_TRAF6_MEDIATED_IRF7_ACTIVATION | 31 | 0.011898 |
| REACTOME_BIOLOGICAL_OXIDATIONS | 156 | 0.012591 |
| REACTOME_RESPIRATORY_ELECTRON_TRANSPORT | 55 | 0.014047 |

## **Supplementary Table 4: Permutation test results for association between the KEGG Arginine & Proline Metabolism Pathway and Systolic Blood Pressure**

| Rank | Permuted P -value |
| --- | --- |
| 1 | 8.55 x 10^-6^ |
| 2 | 1.25 x 10^-5^ |
| 3 | 2.95 x 10^-5^ |
| 4 | 4.03 x 10^-5^ |
| 5 | 5.07 x 10^-5^ |
| 6 | 9.20 x 10^-5^ |
| 7 | 9.49 x 10^-5^ |
| 8 | 0.000103 |
| 9 | 0.000107 |
| 10 | 0.000111 |

- Top 10 lowest p-values observed based on 100,000 permutation tests.

## **Supplementary Table 5: KEGG Arginine & Proline Metabolism Pathway Individual Gene Analyses with Systolic Blood Pressure using UK10K WGS data**

| **Gene** | **# variants** | **UK10K P-value** | **TwinsUK P-value** | **ALSPAC P-value** |
| --- | --- | --- | --- | --- |
| *GOT2* | 3 | 0.01 | 0.08 | 0.05 |
| *PYCR1* | 2 | 0.01 | 0.02 | 0.32 |
| *ALDH1B1* | 3 | 0.03 | 0.29 | 0.09 |
| *NOS1* | 10 | 0.06 | 0.41 | 0.08 |
| *NOS3* | 5 | 0.09 | 0.13 | 0.12 |
| *P4HA2* | 4 | 0.10 | 0.20 | 0.36 |
| *ALDH18A1* | 3 | 0.11 | 0.59 | 0.09 |
| *CPS1* | 13 | 0.13 | 0.07 | 0.65 |
| *ASL* | 2 | 0.15 | 0.93 | 0.07 |
| *ALDH9A1* | 3 | 0.24 | 0.03 | 0.74 |
| *GAMT* | 2 | 0.32 | 0.62 | 0.36 |
| *NAGS* | 3 | 0.33 | 0.35 | 0.76 |
| *P4HA1* | 5 | 0.35 | 0.66 | 0.51 |
| *CKMT2* | 3 | 0.37 | 0.35 | 0.41 |
| *OAT* | 2 | 0.38 | 0.72 | 0.20 |
| *NOS2* | 2 | 0.39 | 0.13 | 0.30 |
| *P4HA3* | 4 | 0.39 | 0.62 | 0.12 |
| *DAO* | 3 | 0.51 | 0.17 | 0.55 |
| *GLUL* | 2 | 0.52 | 0.84 | 0.35 |
| *ALDH4A1* | 5 | 0.52 | 0.55 | 0.62 |
| *GATM* | 2 | 0.62 | 0.67 | 0.80 |
| *GOT1* | 2 | 0.67 | 0.49 | 0.88 |
| *ALDH2* | 3 | 0.75 | 0.11 | 0.87 |
| *ASS1* | 3 | 0.78 | 0.19 | 0.64 |
| *ODC1* | 3 | 0.80 | 0.86 | 1 |
| *GLUD1* | 2 | 0.86 | 0.99 | 0.83 |
| *LAP3* | 8 | 0.87 | 0.28 | 0.75 |
| *ADC* | 2 | 1 | 0.80 | 0.85 |
| *ARG2* | 2 | 1 | 1 | 1 |

- # variants = number of variants analysed, UK10K P-value = SKAT-O p-value for entire UK10K sample, Twins P, SKAT p-value for only TwinsUK individuals , ALSPAC P = SKAT p-value for only ALSPAC individuals p-values according to SKAT-O test, all other genes on this pathway did not contain at least 2 variants after filtering

## **Supplementary Table 6: KEGG Arginine & Proline Metabolism Pathway Individual Gene Analyses with Systolic Blood Pressure in ALSPAC imputed data**

| **Gene** | **# variants** | **P-value** |
| --- | --- | --- |
| *NOS1* | 9 | 0.05 |
| *GLUL* | 2 | 0.23 |
| *GATM* | 2 | 0.25 |
| *LAP3* | 6 | 0.28 |
| *GOT2* | 2 | 0.30 |
| *ADC* | 3 | 0.31 |
| *GAMT* | 2 | 0.38 |
| *ALDH4A1* | 2 | 0.40 |
| *CPS1* | 8 | 0.44 |
| *ASS1* | 3 | 0.57 |
| *P4HA3* | 4 | 0.58 |
| *P4HA1* | 2 | 0.61 |
| *ALDH2* | 2 | 0.71 |
| *PYCR1* | 2 | 0.78 |
| *ALDH9A1* | 2 | 0.84 |
| *NOS2* | 3 | 0.83 |
| *NOS3* | 2 | 0.92 |
| *ALDH18A1* | 2 | 1 |
| *DAO* | 3 | 1 |

# variants = number of variants analysed, P-value = p-values according to SKAT-O test, all other genes on this pathway did not contain at least 2 variants after filtering

## **Supplementary Table 7: Individual SNV results from discovery analysis using UK10K WGS** data

| Gene | Variant Name | HGVSc | Coefficient | P-value |
| --- | --- | --- | --- | --- |
| *ALDH9A1* | chr1:165652241 | c.434C>T | 1.77 | 0.01 |
| *GOT2* | rs149988435 | c.460G>A | -0.65 | 0.01 |
| *ALDH1B1* | rs61741825 | c.1409T>C | -0.30 | 0.02 |
| *PYCR1* | chr17:79899349 | c.-24+823C>G | -0.60 | 0.02 |
| *NOS1* | rs41356652 | c.1783G>A | -0.37 | 0.03 |
| *P4HA1* | rs200482984 | c.*974C>T | 1.15 | 0.05 |
| *P4HA2* | chr5:131607580 | c.78+23291G>A | -1.34 | 0.06 |
| *CPS1* | rs147294932 | c.731G>A | -0.81 | 0.07 |
| *NOS1* | chr12:117655959 | c.4181G>A | 1.28 | 0.07 |
| *P4HA2* | chr5:131607763 | c.78+23108G>A | -1.24 | 0.08 |

- All coefficients and p-values determined by linear regression

## **Supplementary Table 8:** Individual SNV coefficients from Replication analysis using ALSPAC imputed data

| Gene | Variant Name | HGVSc | Coefficient | P-value |
| --- | --- | --- | --- | --- |
| *GATM* | rs146057680_c | c.482A>G | -19.50 | 0.04 |
| *NOS1* | rs76090928_t | c.721G>A | 6.02 | 0.06 |
| *ADC* | rs16835244_t | c.862G>T | 14.00 | 0.13 |
| *P4HA3* | chr11:73965167_c | c.*1318+5801T>G | 14.00 | 0.13 |
| *NOS1* | rs41356652_t | c.1783G>A | 1.97 | 0.13 |
| *ALDH1B1* | rs142427338_t | c.1132C>T | -3.69 | 0.13 |
| *PRODH2* | rs148996461_a | c.968C>T | -7.51 | 0.16 |
| *GLUD1* | chr10:88827840_t | c.721G>A | -7.50 | 0.16 |
| *OAT* | rs121965057_c | c.483C>G | 8.76 | 0.18 |
| *CPS1* | rs147294932_a | c.731G>A | 3.95 | 0.20 |

- All coefficients and p-values determined by linear regression

## **Supplementary Information 3: Evaluation of power from replication analysis**

For this analysis we took all rare variants (MAF≤1%) from within the Arginine and Proline pathway for all individuals in the imputed ALSPAC dataset, where P=0.02 from 71 variants (N=4,380) based on our analysis in the main paper with SBP. We then randomly removed individuals to vary sample size (between n =500 and n = 4,000, using increments of 500) as well as randomly removing variants in this dataset (between 80% to 100% of variants, using increments of 5%). In each possible scenario, variants were reanalysed with SBP, then randomly selecting individuals and variants again for a total of 10,000 permutations and then taking the average power. This allowed us to calculate the relative power using 3 different significance levels (=0.05, 0.10, 0.15) for various scenarios if our sample size and/or number of rare variants had been reduced. The following tables show the results of this analysis, where relative power is drastically reduced as soon as either variable is altered:

| **α = 0.05** | n = 500 | n = 1000 | n = 1500 | n = 2000 | n = 2500 | n = 3000 | n = 3500 | n = 4000 |
| --- | --- | --- | --- | --- | --- | --- | --- | --- |
| % variants = 80% | 0.058 | 0.089 | 0.174 | 0.222 | 0.302 | 0.413 | 0.554 | 0.688 |
| % variants = 85% | 0.060 | 0.093 | 0.177 | 0.230 | 0.322 | 0.432 | 0.561 | 0.727 |
| % variants = 90% | 0.061 | 0.097 | 0.181 | 0.239 | 0.345 | 0.458 | 0.605 | 0.821 |
| % variants = 95% | 0.062 | 0.112 | 0.188 | 0.264 | 0.372 | 0.497 | 0.655 | 0.832 |
| % variants = 100% | 0.064 | 0.121 | 0.198 | 0.319 | 0.426 | 0.541 | 0.702 | 0.914 |

| **α = 0.10** | n = 500 | n = 1000 | n = 1500 | n = 2000 | n = 2500 | n = 3000 | n = 3500 | n = 4000 |
| --- | --- | --- | --- | --- | --- | --- | --- | --- |
| % variants = 80% | 0.080 | 0.155 | 0.254 | 0.323 | 0.453 | 0.598 | 0.703 | 0.832 |
| % variants = 85% | 0.081 | 0.178 | 0.271 | 0.378 | 0.478 | 0.607 | 0.717 | 0.872 |
| % variants = 90% | 0.083 | 0.192 | 0.293 | 0.398 | 0.501 | 0.645 | 0.768 | 0.910 |
| % variants = 95% | 0.088 | 0.209 | 0.305 | 0.446 | 0.537 | 0.687 | 0.803 | 0.941 |
| % variants = 100% | 0.093 | 0.221 | 0.324 | 0.505 | 0.633 | 0.738 | 0.855 | 0.975 |

| **α = 0.15** | n = 500 | n = 1000 | n = 1500 | n = 2000 | n = 2500 | n = 3000 | n = 3500 | n = 4000 |
| --- | --- | --- | --- | --- | --- | --- | --- | --- |
| % variants = 80% | 0.091 | 0.231 | 0.354 | 0.429 | 0.542 | 0.712 | 0.804 | 0.903 |
| % variants = 85% | 0.155 | 0.243 | 0.361 | 0.450 | 0.580 | 0.736 | 0.834 | 0.932 |
| % variants = 90% | 0.178 | 0.268 | 0.378 | 0.489 | 0.612 | 0.761 | 0.863 | 0.959 |
| % variants = 95% | 0.185 | 0.290 | 0.390 | 0.546 | 0.645 | 0.776 | 0.889 | 0.978 |
| % variants = 100% | 0.193 | 0.356 | 0.422 | 0.587 | 0.689 | 0.791 | 0.923 | 0.993 |
